# Clindamycin‐ und Daptomycin‐Versagen bei PVL‐positiver MRSA‐Infektion der Haut und Weichteile

**DOI:** 10.1111/ddg.15988_g

**Published:** 2026-05-05

**Authors:** Michael Zolotas, Kamran Ghoreschi, Ulrike Blume‐Peytavi, Rasmus Leistner, Daniel Humme

**Affiliations:** ^1^ Klinik für Dermatologie Venerologie und Allergologie Charité – Universitätsmedizin Berlin Berlin Deutschland; ^2^ Institut für Hygiene und Umweltmedizin Charité – Universitätsmedizin Berlin Berlin Deutschland

Sehr geehrte Herausgeber,

Panton‐Valentine‐Leukozidin (PVL) ist ein potentes Poren‐bildendes Toxin, das von bestimmten Stämmen von *Staphylococcus aureus* produziert wird, die die lukS‐ und lukF‐Gene tragen. Es ist insbesondere mit erhöhter Virulenz und der Entstehung schwerer Haut‐ und Weichteilinfektionen assoziiert.^1^, ^2^ Das PVL‐Toxin fördert Gewebenekrosen, Leukozytolyse und intensive Entzündungsreaktionen, wodurch entsprechende Infektionen besonders schwierig zu behandeln sind.^1^, ^2^ Der Nachweis von PVL‐bildendem *S. aureus* (PVL‐SA) gelingt durch eine PCR‐Untersuchung der lukS‐ und lukF‐Gene.^3^ Neuere Studien zeigen, dass PVL‐positive Stämme vor allem bei komplizierten Infektionen und bei vulnerablen Patientengruppen wie Kindern häufiger vorkommen. Bei Letzteren sind neben kutanen auch lebensbedrohliche Infektionen wie Osteomyelitis, nekrotisierende Pneumonie oder Fasziitis beschrieben.^4^ Auch junge, gesunde Patienten können wiederkehrende schwere Infektionen durch PVL‐SA erleiden,^5^ die oftmals mit einer Verzögerung von mehreren Monaten diagnostiziert werden.^6^

Wir berichten von einem 69‐jährigen männlichen Patienten ohne relevante Vorerkrankungen oder Immunsuppression, der mit einem ca. 2 cm durchmessenden, erythematös‐indurierten, subkutanen Tumor über der rechten Schulter in unserer Ambulanz vorstellig wurde. Der niedergelassene Dermatologe hatte bereits vor vier Tagen eine Inzision mit Drainage durchgeführt und eine orale Antibiotikatherapie mit Clindamycin (600 mg dreimal täglich) eingeleitet. Eine weitere tiefe Inzision erfolgte bei uns unter lokaler Anästhesie mit Abnahme von Eitermaterial zur mikrobiologischen Untersuchung. Zu diesem Zeitpunkt zeigte sich keine Leukozytose und das CRP war leicht erhöht auf 24,5 mg/L [Norm: < 5 mg/L]. Die orale Clindamycin‐Therapie wurde bis zum Vorliegen des Antibiogramms fortgeführt.

Drei Tage später kam es zu einer Verschlechterung mit Vergrößerung des subkutanen Tumors auf ca. 5 cm. Laborwerte zeigten einen CRP‐Wert von 90 mg/L und eine Leukozytose von 13,6/nL [Norm: 3,9–10,5/nL] mit Neutrophilie von 10,8/nL [Norm: 1,5–7,7/nL]. Kulturergebnisse bestätigten einen methicillinresistenten *Staphylococcus aureus* (MRSA) mit Clindamycin‐Resistenz. Daraufhin wurde der Patient stationär aufgenommen und eine intravenöse Vancomycin‐Therapie (1,5 g zweimal täglich) entsprechend dem Antibiogramm (ergänzende online Tabelle S1) zusammen mit Dekolonisationsmaßnahmen eingeleitet. Sonographisch zeigte sich eine ca. 2 cm tiefe Läsion mit heterogener Echogenität, jedoch ohne eindeutige Abszessformation. Im Laufe der nächsten zwei Tagen wurden Vancomycin‐Spiegel wiederholt als unzureichend ermittelt, zudem verschlechterten sich das Hautbild (Abbildung 1a) und die Entzündungsparameter (CRP: 190 mg/L, Leukozyten: 11,4/nL), sodass die Antibiose auf Daptomycin (10 mg/kg einmal täglich) umgestellt wurde, wogegen der Erreger gemäß Antibiogramm sensibel war.

**ABBILDUNG 1:**
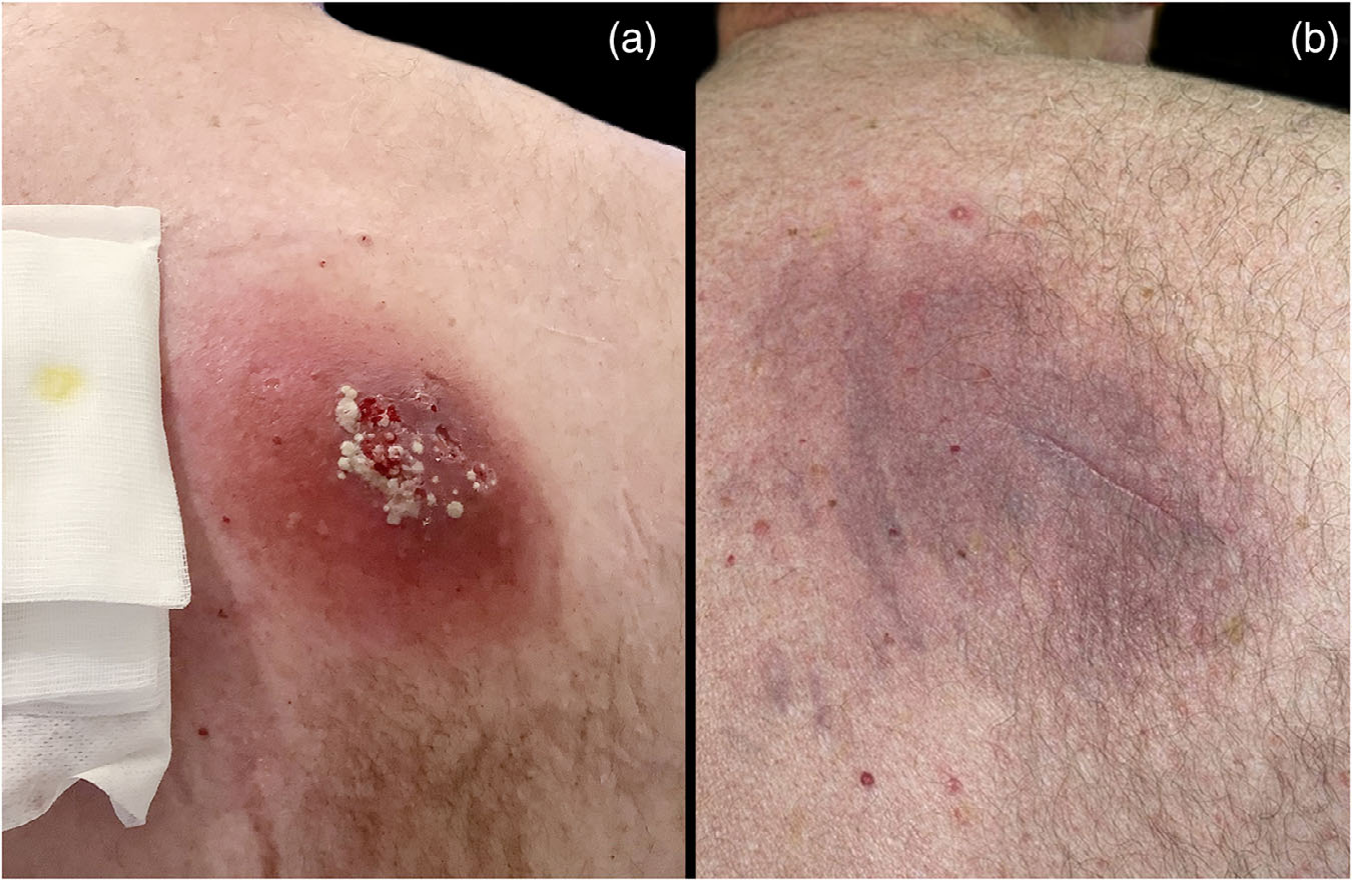
Fotodokumentation des Patienten: (a) zwei Tage nach stationärer Aufnahme; (b) zwanzig Tage nach Entlassung.

Die am Folgetag darauffolgende tiefe Inzision und Drainage zeigten leichte Besserung unter Daptomycin, jedoch persistierte die purulente Sekretion. Eine wenige Tage später durchgeführte MRT‐Untersuchung zeigte eine ca. 10 cm durchmessende und 2,5 cm tiefe entzündliche Raumforderung mit ausgeprägtem umgebendem Ödem bis zur Faszie des Musculus trapezius, jedoch ohne intramuskuläre Beteiligung (Abbildung 2). Trotz fortgesetzter Therapie blieben die Entzündungsparameter über die nächsten 18 Tage erhöht, und aus der Wunde konnte weiterhin MRSA kultiviert werden. Eine weitere MRT‐Untersuchung zeigte nur minimale Rückbildung bei persistierender Läsion.

**ABBILDUNG 2:**
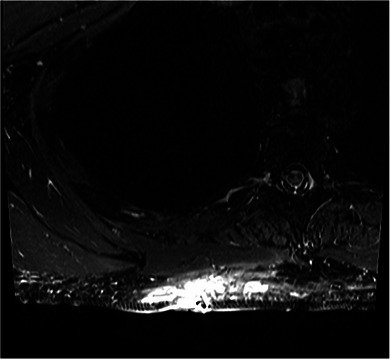
Beispielsequenz der MRT‐Untersuchung neun Tage nach stationärer Aufnahme.

Nach dreiwöchiger Daptomycin‐Therapie zeigte sich in der mikrobiologischen Untersuchung weiterhin MRSA‐Wachstum im kutanen Tumor; zudem bestätigte eine PCR‐Untersuchung die PVL‐Produktion vom MRSA. Nach infektiologischer Rücksprache wurde die antibiotische Therapie auf intravenöses Co‐Trimoxazol (960 mg) und Rifampicin (450 mg) jeweils zweimal täglich umgestellt. Dieses Therapieschema führte zu einer deutlichen klinischen Besserung, sodass der Patient nach neun Tagen entlassen wurde und eine weitere Woche oral mit Co‐Trimoxazol behandelt wurde. Kontrollkulturen sowie wiederholte Abstriche aus Nasenrachen‐ und Wundregion waren negativ. Die Wundheilung war vollständig (Abbildung 1b) und ein Rezidiv trat nicht auf.

Dieser Fall verdeutlicht die therapeutische Herausforderung durch PVL‐MRSA – einen hochvirulenten und zunehmend häufigeren Erreger.^7^ Die Behandlung gestaltete sich insbesondere aufgrund der Clindamycin‐Resistenz sowie eines ungewöhnlichen klinischen Versagens von Daptomycin trotz in‐vitro‐Suszeptibilität schwierig. Clindamycin‐Resistenz bei MRSA ist nicht selten und teilweise induzierbar, was unerkannt zu Therapieversagen führen kann.^8^ Daptomycin‐Resistenz ist hingegen selten,^9^ und besonders in vorliegender Kombination mit PVL‐Produktion mit erhöhter Virulenz assoziiert.

Der klinische Verlauf mit insgesamt mehr als vier Wochen stationärer Behandlung unterstreicht die Bedeutung einer frühzeitigen und genauen Diagnostik mit fortlaufender mikrobiologischer Kontrolle, einschließlich wiederholter Kulturen und Antibiogramme – nicht nur zu Beginn, sondern auch während der Therapie bei persistierenden Symptomen. Darüber hinaus reflektiert der Fall die globale und zunehmende Problematik der antimikrobiellen Resistenz,^10^ und betont die Dringlichkeit maßgeschneiderter Antibiotikatherapien und der Einhaltung standardisierter Behandlungsprotokolle bei resistenten Erregern.

## DANKSAGUNG

Open access Veröffentlichung ermöglicht und organisiert durch Projekt DEAL.

## INTERESSENKONFLIKT

Keiner.

## Supporting information



Supporting Information
