# Supplementary material for: Clindamycin‐ und Daptomycin‐Versagen bei PVL‐positiver MRSA‐Infektion der Haut und Weichteile
Source: J Dtsch Dermatol Ges. 2026 May 5;24(5):674–6. [Article in German] doi: 10.1111/ddg.15988_g (PMC13140130; doi:10.1111/ddg.15988_g)
Supplement: Supplementary file 1 — Supporting Information [file DDG-24-674-s001.docx]

**SUPPLEMENT TABELLE 1**

| Antibiotikum | Ergebnis | MHK |
| --- | --- | --- |
| Penicillin | R | ≥ 0.5 |
| Ampicillin | R |  |
| Oxacillin | R | ≥ 4.0 |
| Ampicillin/Sulbactam | R |  |
| Piperacillin | R |  |
| Piperacillin/Tazobactam | R |  |
| Cefazolin | R |  |
| Cefuroxim | R |  |
| Ceftriaxon | R |  |
| Imipenem | R |  |
| Meropenem | R |  |
| Levofloxacin | I | 0.25 |
| Gentamicin | S | ≤ 0.5 |
| Tobramycin | S |  |
| Teicoplanin | S | ≤ 0.5 |
| Vancomycin | S | 1.0 |
| Erythromycin | R | ≥ 8.0 |
| Clindamycin | R | ≥ 4.0 |
| Doxycyclin | R | ≥ 16.0 |
| Tigecyclin | S | ≤ 0.12 |
| Linezolid | S | 1.0 |
| Daptomycin | S | 0.25 |
| Fosfomycin | S | ≤ 8.0 |
| Fusidinsäure | S | ≤ 0.5 |
| Mupirocin | S | ≤ 1.0 |
| Rifampicin | S | ≤ 0.03 |
| Cotrimoxazol | S | ≤ 10.0 |

Supplementary Table 1: Antibiogramm vom PVL-MRSA, der in der Probe vom 19. Januar 2024 nachgewiesen wurde (R: resistent; I: sensibel bei erhöhter Exposition; S: sensibel unter Standarddosis; MHK: minimale Hemmkonzentration in mg/l – Die Interpretation der MHK-Werte erfolgt nach EUCAST-Standard 13.0)
